# Treatment progress of cryptozoospermia with Western Medicine and traditional Chinese medicine: A literature review

**DOI:** 10.1002/hsr2.1019

**Published:** 2022-12-27

**Authors:** Huang Liu, Zefang Luo, Jinghua Chen, Houbin Zheng, Qingqi Zeng

**Affiliations:** ^1^ The First School of Clinical Medicine Nanjing University of Chinese Medicine Nanjing China; ^2^ NHC Key Laboratory of Male Reproduction and Genetics, Department of Andrology, Guangdong Provincial Reproductive Science Institute (Guangdong Provincial Fertility Hospital) Human Sperm Bank of Guangdong Province Guangzhou China; ^3^ Reproductive Medical Centre of Sun Yat‐sen Memorial Hospital Sun Yat‐sen University Guangzhou China; ^4^ Department of Integrated Chinese and Western Medicine Jiangsu Health Vocational College Nanjing China

**Keywords:** cryptozoospermia, mechanisms, traditional Chinese medicine, treatment, Western Medicine

## Abstract

**Background and Aims:**

Cryptozoospermia is an extreme oligozoospermia with an unsatisfactory treatment effect, with an incidence rate of approximately 8.73% in male infertility, whose effective solution has become the call of the times. Western Medicine has achieved certain effects through drugs, surgery, and assisted reproductive therapy, but this is still not ideal. Traditional Chinese medicine (*TCM*) has made many achievements in other disciplines; however, there is still a lack of evidence‐based medical evidence to improve sperm production.

**Methods:**

The relevant literatures from the China National Knowledge Internet (*CNKI*) and PubMed in the past 10 years were collected in this article, of which the mechanisms, advantages, or current controversies of various treatment methods of Western Medicine and *TCM* were analyzed, to find new treatment methods and research directions.

**Results:**

With the development of modern science and technology, medical treatments for cryptozoospermia have become increasingly abundant; however, there is still no universally recognized unified and effective guiding plan. Although *TCM* has not been fully verified by evidence‐based medicine, most *TCM* combined with Western Medicine can achieve unexpected results.

**Conclusion:**

The combination of *TCM* and Western Medicine may become a bane for cryptozoospermia and bring good news to infertile men worldwide.

## 1 INTRODUCTION

According to the World Health Organization (*WHO*), couples who lived together for 1 year, had a normal and regular sex life, and could not conceive without taking any contraception were called infertile, with a worldwide incidence of approximately 15%–20%.^1^ Male infertility, which is caused solely by male factors, accounts for approximately 50% of infertility cases.^2^ Abnormal sperm is the primary cause of male infertility. Cryptozoospermia is extremely difficult to treat, and has rarely been studied.

Based on *WHO* standards, cryptozoospermia was defined as the absence of sperm in the ejaculated semen by microscopic examination, but was present in the centrifuge sediment.^3^ Cryptozoospermia, also known as cryptozoospermic, is a special type of extreme oligospermia, with an incidence of approximately 8.73%.^4^ Owing to its unique clinical manifestations, it is easily misdiagnosed as azoospermia. The unsatisfactory effect of this treatment confuses many doctors worldwide.

At present, all factors causing decreased spermatogenesis may be the mechanism of cryptozoospermia, such as chromosomal abnormalities,^5^ genetics,^6^ urogenital system congenital anomalies,^7^ infection,^8^ immune damage,^9^ testicular microlithiasis,^10^ hydrocele,^11^ varicocele,^12^ abnormal sex hormones,^13^ toxins,^14^ radiation,^15^ and tumors.^16^

With the development of cryotherapy, biological treatment, drugs, surgery, assisted reproductive technology (*ART*), and traditional Chinese medicine (*TCM*), an increasing number of studies or case reports of new methods have obtained good results; however, there is no consensus, and more uncertainties need to be discussed. We divided these treatments into three parts: Western Medicine: methods to improve sperm counts, Western Medicine: methods for the use of sperm, TCM: holistic concept, and review the relevant literature to find new treatment methods and research directions.

## 2 MATERIALS AND METHODS

The relevant literatures from the China National Knowledge Internet (*CNKI*) and PubMed from January 1, 2012 to December 31, 2021 were collected, with the keywords of “Cryptozoospermia,” “Cryptozoospermic,” “Spermatogenesis,” “Traditional Chinese Medicine,” “Hormone,” “Treatment” and “Assisted reproduction,” of which the mechanisms, advantages, or current controversies of various treatment methods of Western Medicine and *TCM* were analyzed, to find new treatment methods and research directions. The inclusion criteria was: With complete data information, the treatment and intervention plans including cryptozoospermia. The exclusion criteria was: Review articles, animal experiments, comments of articles and incomplete information. A total of 116 articles were related to cryptospermia, including 100 articles from PubMed and 16 articles from CNKI. Finally, after deleting repetitive articles, 64 articles were included according to relevant standards.

## 3 RESULTS

### 3.1 Western Medicine: Methods to improve sperm count

**Figure 1:**
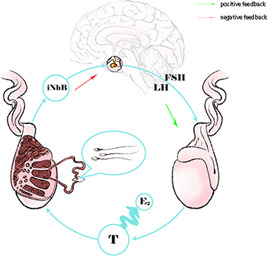
Endocrine regulation mechanism of modern drug therapy. E_2,_ estradiol; FSH, follicle stimulating hormone; Green arrows, positive feedback; iNhB, inhibin B; LH, luteinizing hormone; Red arrows, negative feedback; T, testosterone.

Currently, there is no clear or effective treatment for cryptozoospermia. A large number of small samples or single‐center clinical observations have found that hormonal drugs, estrogen receptor antagonists, antioxidants, energy metabolism, or trace element supplementation could achieve certain therapeutic effects,^17^, ^18^ but some have also held opposing views.^19^ Although the effectiveness of drug treatment for spermatogenesis is slower than that of surgical treatment, it is more cost‐effective and acceptable to patients. The main theoretical basis for methods to improve sperm count is the positive and negative feedback mechanisms of endocrine regulation (Figure 1). Therefore, drug therapy has always been the first choice of treatment for cryptozoospermia.

#### 3.1.1 Exogenous hormone therapy

Exogenous hormone therapy is mainly based on the positive and negative feedback regulatory mechanisms of reproductive hormones. Testosterone (*T*) is considered an endogenous driving force that directly enhances spermatogenesis. Follicle stimulating hormone (*FSH*) and luteinizing hormone (*LH*) regulate the concentration of testosterone directly or indirectly and affect the initiation of the mitotic and meiotic cycles of spermatogonial cells in spermatogenic tubules.^20^ Therefore, exogenous supplementation with a sufficient amount of endogenous hormones or inhibition of their degradation rate should effectively regulate spermatogenesis. However, long‐term clinical observations have shown that high concentrations of testosterone also inhibit spermatogenesis,^21^, ^22^ which is most common in patients undergoing testosterone replacement therapy.^23^ Patients with secondary cryptorzoospermia induced by testosterone replacement therapy were observed, and the results showed that the serum hormone levels and sperm concentration could return to normal ranges approximately 6–12 months after withdrawal of testosterone, suggesting that if men are trying to conceive, they should be prohibited from testosterone therapy.^17^, ^24^ Therefore, most current academic guidelines do not recommend the use of high‐dose long‐term testosterone as a stimulant for sperm production.^25^, ^26^

*FSH* directly acts on Sertoli cells, increasing the concentration of testosterone binding protein and producing inhibin B, which inhibits testosterone decomposition.^27^ Sperm regulation is also believed to be closely related to testosterone. Exogenous injection of *FSH*, the most common of which is human menopausal gonadotropin (*HMG*), is commonly used as a first‐line empirical drug to improve spermatogenesis and has been recognized by expert consensus or diagnosis and treatment guidelines.^28^ Currently, *HMG* has been widely reported to improve spermatogenesis in men with nonobstructive azoospermia to obtain ejaculated sperm or improve the probability of sperm finding during microtesticular sperm extraction.^29^ However, the efficacy of this technique on cryptorzoospermia is not optimistic and has rarely been reported. This led researchers to question whether cryptorzoospermia is different from nonobstructive azoospermia, whose therapeutic target was elusive.

*LH* acts directly on the interstitial cells and promotes testosterone secretion. Exogenous *LH* supplementation, which most commonly involves human chorionic gonadotropin (*HCG*), is the most common method for regulating spermatogenesis and addresses the needs of a large proportion of patients with hypergonadotropic hypogonadism.^30^ However, with the application of *HCG* and an expanded observation sample, its efficacy has also been challenged. Especially for sports patients, *HCG* was listed as a banned substance for male athletes by the World Anti‐Doping Agency (*WADA*); therefore, it has the potential to become a focus of debate and the beginning of conflicts between doctors and patients.^31^ The use of *HCG* was also monitored because of the regulatory regulations for drug use.^32^ There was also concern about the appropriateness of exogenous *HCG* use when *LH* was elevated and sperm was decreased.^33^ When the body's negative feedback regulation fails, exogenous *HCG* supplementation in patients with elevated *LH* or *FSH* levels does not seem to be effective in reducing endogenous hormone levels. When the negative feedback regulation mechanism is normal, exogenous supplementation often suppresses endogenous *FSH* and *LH* to extremely low levels, which induces idiopathic hypogonadotropic hypogonadism.^34^

However, exogenous hormone treatment still needs further discussion, especially whether low‐dose hormones could promote sperm production might become a new direction of current research, which is worthy of more large‐sample, multicenter observation and follow‐up.

#### 3.1.2 Antagonist therapy

Estrogen receptor antagonists had been shown to inhibit testosterone degradation to ensure high levels of testosterone in spermatogenic tubules and promote spermatogenesis. Currently, there are two commonly used of selective estrogen receptor modulators (SERM). The use value of these drugs were uneven, and the recommendation degree of each drug differed according to the guidelines and expert consensus.^35^, ^36^ The effects of clomiphene were initially recognized by many researchers,^37^ but the side effects of high doses of clomiphene were later thought to be adverse to sperm, possibly increasing sperm count rather than quality. Clomiphene was also reported to have an unsatisfactory outcome in the treatment of secondary cryptozoospermia caused by testosterone replacement therapy.^23^ The use of tamoxifen changed this view: tamoxifen was ideal for sperm quality improvement, but there were also deficiencies. When the concentration was too high, tamoxifen inhibited spermatogenesis in zebrafish, causing spermatogenesis disorders.^38^

In recent years, letrozole, an aromatase inhibitor, has been shown to improve sperm parameters. However, there are data showing that it does not work well for cryptozoospermia. A clinical trial was conducted on 86 patients with oligospermia (including cryptozoospermia) who were treated with letrozole. They found that although *T* increased and *E*_*2*_ decreased in 95.3% of patients after treatment, the sperm concentration and total motility count improved in only 18 oligozoospermic men with hypoandrogenism, but did not improve in cryptozoospermic patients.^19^

Therefore, the use of hormone antagonists remains a controversial topic. There are few studies and no unified guidelines for these, especially their indications, and the stage of hormone at which levels would indicate a better therapeutic effect pretreatment.

#### 3.1.3 Metabolic regulators

Metabolism regulators can be regarded as a basic therapy for sperm.^39^ It mainly includes oxidation treatment, improved energy mixing, and microcirculation, which have received increasing attention and arguments.^40^ The main principle of oxidation treatment is to improve the cell oxidative stress state and is considered to improve spermatogenesis in prime movers. The most common ones were vitamins E and C, but this still needs to be confirmed by large samples and multicenter data.^41^, ^42^ Drugs commonly used to improve energy metabolism include coenzyme Q_10_ and l‐carnitine, which are often combined with antioxidant effects.^43^

The latest review of 61 included studies indicated that there was insufficient evidence that empiric therapy and nutritional supplements improved semen parameters; moreover, there was insufficient evidence that these methods increased live birth or pregnancy rates.^44^ Increasing evidence suggests that improved microcirculation perfusion of the testis can effectively promote spermatogenesis.^45^ Therefore, drugs that were used to improve microcirculation for cardiovascular disease have also been used in patients with low sperm production function,^46^, ^47^ and their targets were mainly capillary endothelial cells, but there was a lack of strict evidence‐based medical evidence to prove their effectiveness.

The idea that energy compounds boost sperm count is emerging as a challenge to conventional wisdom. For example, the conventional wisdom was that exercise boosted sperm counts but depleted the body's capacity. Therefore, exercise boosted sperm production, or did it expend energy and reduce sperm count. Whether exercise can improve sperm count has become a controversial topic.

### 3.2 Western Medicine: Methods for the use of sperms

**Figure 2:**
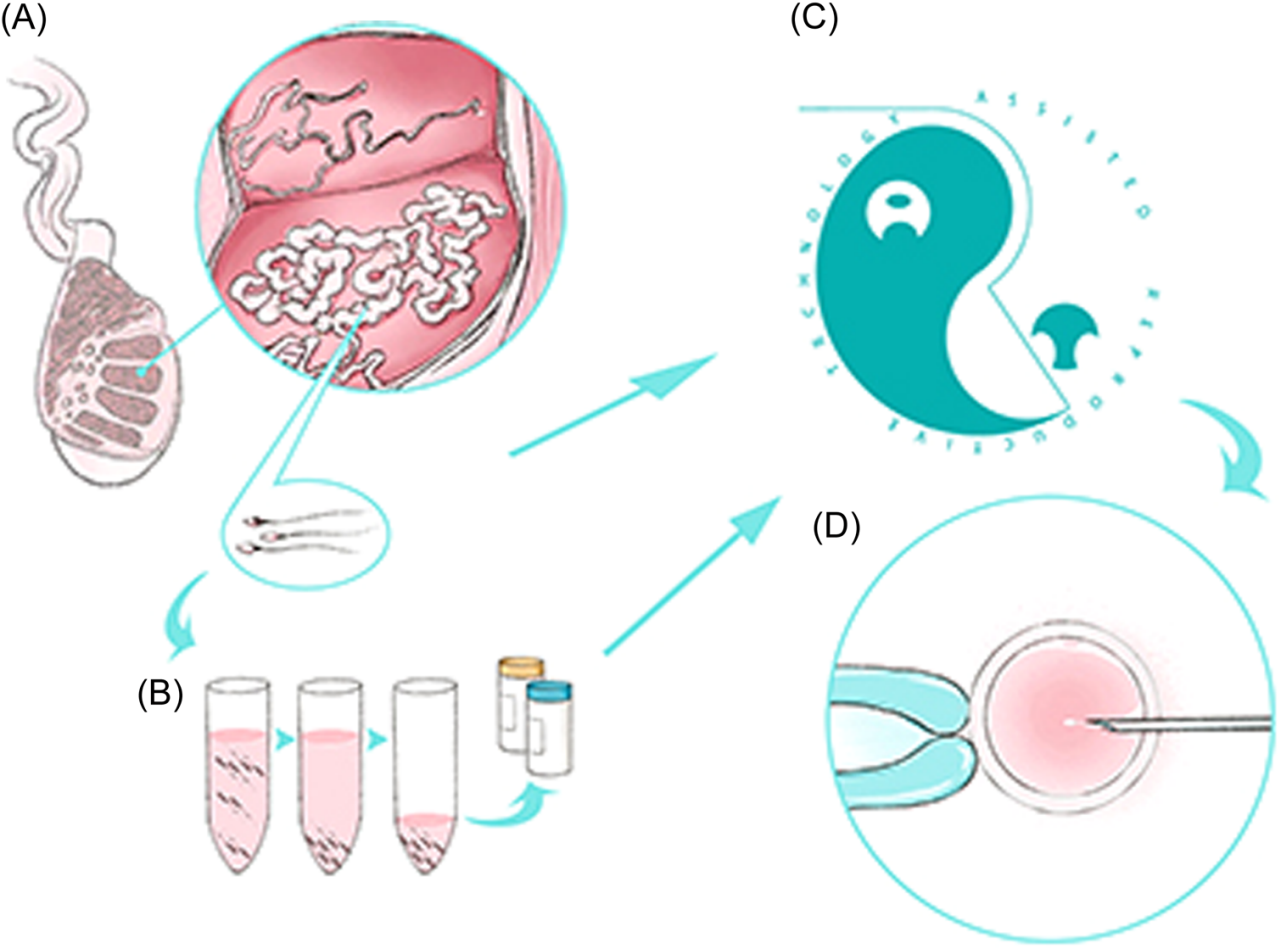
Modern mainstream methods of sperm recovery and use (A) microsurgical sperm extraction of testis; (B) sperm cryopreservation; (C) ART, assisted reproductive technology, (D) ICSI, intracytoplasmic sperm injection.

In Western Medicine, the effective use of a small number of cryptozoospermic patients is controversial. After obtaining as many sperm as possible, how to make good use of them from different sources and improve the pregnancy rate was the bottleneck that limited the success rate of cryptozoospermia patients in Western Medicine. Currently, surgery, cryopreservation, and *ART* are the mainstream methods for using sperm for cryptozoospermia (Figure 2).

#### 3.2.1 Microsurgery: Sperm recovery for ART

Studies have shown that surgery is more cost‐effective for infertile men.^48^ With the use of microsurgery, an increasing number of cryptozoospermia patients are believed to be able to remove the cause and improve spermatogenesis or obtain more sperm for assisted reproduction. Among these, varicocele surgery and testicular microsurgery are representative of their applications.

The efficacy of varicocele surgery is controversial. Although varicocele surgery could significantly improve sperm quality, but that was still doubted hard to improve the final pregnancy rate. Some authors argued that this was an unreasonable evaluation because no one could guarantee that all the patients' spouses were the same. Therefore, we thought the improvement of sperm quality was the final value performance of the effect of varicocele surgery. In particular, the safety and effectiveness of varicocele surgery under a microscope are significant. The latest two review articles suggest that microscopic varicocele surgery can not only improve the pregnancy outcome of patients with oligoasthenospermia^49^ but also improve the fertility outcome of patients with non‐obstructive azoospermia. A total of 27.3% of patients may find sperm in ejaculated semen 10.5 months after operation.^50^ A previous study found that cryptozoospermia with varicocele achieved good results after microscopic surgical treatment.^51^

Microscopic sperm extraction (*M‐TESE*) (Figure 2A) was also considered the ideal technique to obtain sperm, which combined with ART could achieve the best effect at present, as it could effectively identify local spermatogenic foci in the testicle and solve the problem of most infertile men. *M‐TESE* successfully recovered sperm in 58% of men with cryptozoospermia (≥5 sperms recovered during the procedure).^52^ Studies have shown that testicular sperm aspiration (*TESA*) and *M‐TESE* have the same effect on sperm retrieval in men with severe oligospermia, but *M‐TESE* has a significantly higher sperm recovery rate than *TESA* in men with cryptozoospermia.^53^ A study performed testicular sperm extractions in 56 men with cryptozoospermia and found that 88% of the men (42/48) who underwent *M‐TESE* and 25% of the men (2/8) who underwent *TESA* had successful sperm retrieval.^54^ In addition, 96% of the cryptozoospermic men (23/24) who underwent *M‐TESE* could receive sperm from the testis, but only 43% of the men (3/7) could receive sperm who underwent *TESA*, and the pregnancy rates of intracytoplasmic sperm injection (*ICSI*) in these groups could reach 33% (6/18). Thus, *M‐TESE* is a successful sperm extraction technique for men with cryptozoospermia.^55^

#### 3.2.2 Cryotherapy: Sperm freezing for ART

The number of sperm in cryptozoospermic patients is extremely low, and it is impossible to achieve a natural pregnancy. Currently, the method of conception depends on *ART*. In particular, *ICSI* is the ultimate and most effective reproductive choice for patients with cryptozoospermia, but the success rate is often limited by the sperm source.^56^

Cryopreservation (Figure 2B) of spermatozoa is considered the most effective way to solve the reproductive needs of patients with cryptozoospermia, which is characterized by the presence of spermatozoa in the centrifugal sediment of ejaculated semen. The recovery and use of spermatozoa frozen in Cell Sleepers is simpler and more effective than traditional methods,^57^ there was no need to perform any sperm retrieval procedure on the day of oocyte collection,^58^ and it was more effective in cryopreserving sperm samples from men with severely reduced fertility.^59^ Many researchers have also found that cryopreservation of a single sperm could provide more advantages.^60^, ^61^ One review of the advantages and disadvantages of cryopreservation of single sperm and its clinical application outcomes from the perspectives of cryopreservation carriers, freezing procedures, and cryoprotectant formulas showed that cryopreservation of single sperm could benefit patients with cryptozoospermia.^62^

For patients with cryptozoospermia, fresh ejaculated sperm or sperm in the testis or frozen sperm results in a higher success rate of *ART*. There were many different opinions and it was difficult to form a unified view. Bendikson et al.^63^ compared the *ICSI* pregnancy outcomes of 16 men with cryptozoospermia and found that the success rate of *ICSI* with testicular sperm was significantly higher than that with ejaculated sperm (50.0% vs. 14.3%). Rong et al.^64^ also performed *ICSI* in 13 male couples with cryptozoospermia but found that sperm obtained from fresh testicular puncture had a better implantation rate than frozen testicular sperm and ejaculated sperm (65.3% and 53.2%, *p* < 0.05).

The fertilization rate of frozen‐thawed ejaculated sperm was reported to be lower than that of fresh ejaculated sperm in patients with cryptozoospermia.^65^ However, another study denied this view; they analyzed the outcomes of 83 *ICSI* cycles in 55 men with cryptozoospermia and found that there was no relationship between the fertilization ability of thawed sperm and the parameters before freezing or the source of sperm. They were also convinced that it was a rare event that failed to obtain sperm in patients with cryptozoospermia whose ejaculated sperm were frozen.^66^

An epidemiological study showed that, by retrospectively analyzing the treatment of 169 patients with severe oligospermia between 2004 and 2011, only 5.9% of 84 patients who had frozen sperm before *ICSI* chose to use frozen sperm for *ICSI*, and the outcome of *ICSI* was not significantly different from that of men with fresh ejaculated sperm. Moreover, the proportion of cryopreserved sperm before *ICSI* was high in patients with severe oligozoospermia and cryptozoospermia, but the utilization rate of these cryopreserved sperm in actual *ICSI* cycles was very low, which would greatly reduce the value of sperm cryopreservation in clinical practice.^67^

#### 3.2.3 ART: Use of sperm

For cryptozoospermia patients, *ART* (Figure 2C) was considered the ultimate treatment in Western Medicine, especially *ICSI* (Figure 2D), which was pinned on high expectations. However, the source of sperm that can achieve the best success rate remains a controversial issue. Most researchers believe that fresh spermatozoa from testicular punctures should be the first choice for *ICSI*. Ido et al. analyzed 116 *ICSI* cycles in 17 cryptozoospermia patients and found that *TESE*‐derived sperm showed much higher implantation rates, pregnancy rates, and infant birth rates (20.7% vs. 5.7%, 42.5% vs. 15.1%, 27.5% vs. 9.4%) than ejaculated sperm.^68^

Xie and colleagues performed *ICSI* on 35 patients with cryptozoospermia and found that the normal fertilization rate of patients with epididymal sperm aspiration (*PESA*) (75.6%) was significantly higher than that of ejaculated sperm (62.1%) and *TESA* sperm (61.6%). However, there were no significant differences in the high‐quality embryo rate, implantation rate, or clinical pregnancy rate among the three groups (*p* > 0.05). Although the sperm obtained by *PESA* could achieve a higher normal fertilization rate, there was no significant effect on embryo quality and developmental potential in *ICSI* cycles. Even the source of sperm plays a leading role in embryonic development.^69^

A study of five cohort studies, with 272 *ICSI* cycles and 4596 oocytes, also found that there was no difference in *ICSI* pregnancy rate between testicular sperm and ejaculated sperm in patients with cryptozoospermia. The current literature does not support the recommendation that testis sperm should be used preferentially in *ICSI* in cryptozoospermic men.^70^

However, Amirjannati and colleagues considered that the low sperm concentration in the ejaculated semen of men with cryptozoospermia was a significant factor limiting the effectiveness of their treatment. Comparing 19 *ICSI* cycles with testicular sperm to 208 *ICSI* cycles with ejaculated sperm, the fertilization rates of testicular sperm and ejaculatory sperm were similar (60% and 68%, respectively; *p* ≥ 0.05). In light of the invasive nature of *TESE* sperm retrieval, ejaculated sperm should be recommended over testicular sperm whenever possible in patients with cryptozoospermia.^53^

### 3.3 *TCM*: Holistic concept

Due to historical conditions, there was no name of “cryptorzoospermia” in the classics of *TCM*, but predecessors should have known about cryptorzoospermia for a long time. Throughout the *TCM* classics, ancient Chinese physicians had similar descriptions, which should be equivalent to the categories of “jing leng” (cold sperm), “jing shao” (less sperm), and “jing xi” (thin sperm).^71^, ^72^, ^73^ The treatment of spermatogenesis in *TCM* involves two methods: internal and external. The internal treatment was mainly drug therapy, including Chinese herbal medicine or patent medicine, represented by decoctions, granules, capsules, and tablets. The external treatment was mainly acupuncture, including acupuncture and moxibustion, and topical treatment. *TCM* believes that cryptorzoospermia is closely related to the “shen” (kidney), “pi” (spleen), “wei” (stomach), and “gan” (liver). The main causes are deficiency, excess, cold, heat, phlegm, blood stasis, and depression in the kidney, liver, and spleen. Individualized treatment based on different theoretical viewpoints often achieves ideal results.

#### 3.3.1 Internal treatment

*TCM* internal treatment is based on oral medicine, including classical Chinese herbal decoction and *TCM* granules, tablets, or capsules. Prescriptions and decoctions have always achieved surprising results.^74^ Although their unique ingredients have not been completely separated and identified, they have remarkable functions.^75^ The convenience of Chinese patent medicine made it a substitute for decoction and prescription, which was considered to be the product of the combination of *TCM* and Western Medicine, and laid the foundation for entering the modern pharmaceutical market.

**Figure 3:**
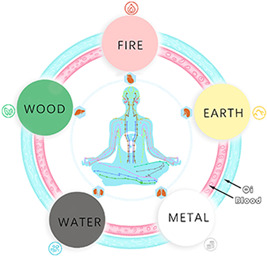
Theoretical basis of internal treatment of traditional Chinese Medicine

*TCM* believed that the human body was an effective balance cycle, and the theoretical basis of internal treatment of *TCM* was to balance *Yin‐Yang* and *Wu‐Xing* (*Five‐Elements)*, regulate *Qi* and *Blood* circulation, clear the *Jing (Channels)* and activate *Luo (Collaterals)*, and finally recover the native functionality of *Zang‐Fu (Organs)*. With the dialectical analysis, *TCM* experts provided personalized treatment to restore the overall function of the patients, such as warm for cold or pour for full (Figure 3).

In recent years, there has been an increasing number of clinical studies on *TCM*,^76^, ^77^, ^78^ and randomized controlled trials^79^, ^80^, ^81^ have also been carried out one after another, which achieved certain effects and were increasingly recognized by Western Medicine. Therefore, the *TCM* decoction plays an important role in improving the number of sperm. In the fields of network pharmacology,^78^, ^82^ animal experiments,^81^, ^83^, ^84^ and human experiments,^79^, ^80^, ^85^, ^86^ it could be used as a potential development object for cryptorzoospermia.

A meta‐analysis of 1488 patients from 14 *RCTs* showed that the total effective rate of patients treated with a combination of *TCM* and vitamins was better than that of patients treated with vitamin *E* or vitamin *E* + *C* alone. The differences in sperm density, motility, and motility among the three groups after treatment were significant. *TCM* combined with vitamins effectively improves sperm count and motility and plays a role in spermatogenesis and fertility.^87^

#### 3.3.2 External treatment

**Figure 4:**
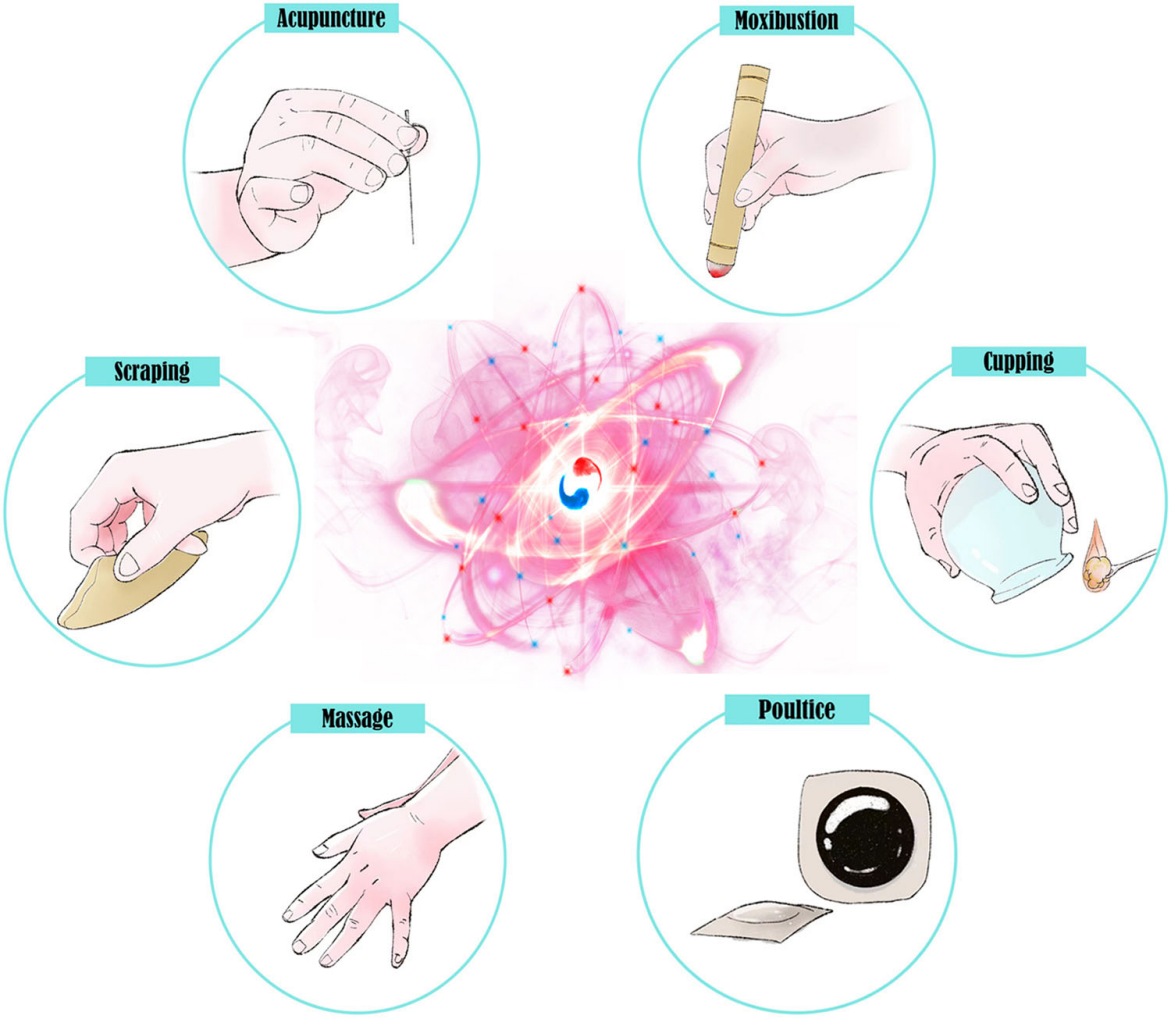
Theoretical basis of external treatment of traditional Chinese Medicine

As a representative of Orientalism and the treasure of the cultural heritage of China, external treatment played an important role in *TCM*. External treatment mainly includes acupuncture,^88^ scraping, cupping, moxibustion,^89^ massage, sticking, and other methods. Its main mechanism is to stimulate local tissues of the human body, promote the circulation of *Blood‐Qi*, dredge *Channels‐Collaterals*, eliminate obstruction and siltation, and activate the systemic immune system to achieve the purpose of treat diseases (Figure 4).

##### Acupuncture

Acupuncture was used to pierce the needle into the body at a certain angle and to stimulate the meridians and acupoints by lifting, inserting, twisting, and turning to achieve the effect of dispelling wind, cooling, dehumidification, and relieving pain.^64^, ^90^, ^91^

With the development of modern science and technology, electroacupuncture has been produced by the combination of traditional acupuncture and Western Medicine, and has been widely applied in China. A randomized controlled study showed electroacupuncture could increase seminal plasma zinc, NAG, and fructose levels in the semen, affecting sperm parameters.^92^ This effective combination of traditional ethnic and Western Medicine maximized the therapeutic effect of both, which has great potential for the development of *TCM*. Animal experiments suggested that electroacupuncture stimulation of ST36 (*Zusanli*) could also improve the function of Sertoli cells^93^ and reduce damage to Sertoli cytoskeleton proteins.^94^ Systematic reviews and meta‐analyses have demonstrated that acupuncture is effective in improving sperm motility.^95^, ^96^, ^97^ However, its effect on sperm count requires further clinical evidence.

Scholars from other countries also found that acupuncture could improve reproductive hormone function^91^ and promote sperm production in patients with nonobstructive azoospermia.^98^ Therefore, in view of the emergence of these cases, we considered that acupuncture might have many undiscovered functions, which had brought us significant inspiration, and that the application of acupuncture in cryptorzoospermia might be a worthy attempt to expand.

##### Moxibustion

Moxibustion was made of artemisia argyi as raw material and then lit and baked at specific acupoints of the human body to keep the heat generated by the burning of artemisia argyi entering the body to achieve the functions of promoting blood circulation and removing stasis, dispelling wind and cold, reducing swelling and relieving pain, and reinforcing *Yang‐Qi*.

Moxibustion is often combined with acupuncture. Some randomized controlled studies have shown that moxibustion combined with drugs^89^ or moxibustion combined with electroacupuncture^99^ might improve sperm motility and concentration. Animal experiments have suggested that electroacupuncture stimulation of ST36 (*Zusanli*) and BL23 (*Shenshu*) could improve the expression of androgen receptors.^100^

##### Guasha (*Scrapping*)

Guasha is an ancient *TCM*, and scrapping board and scrapping oil were used in the relevant parts of the skin repeatedly scraped to dredge the meridians, blood circulation, and stasis.^101^

Studies have shown that guasha can improve local blood circulation and reduce breast swelling.^102^ The main mechanism may be related to the promotion of parasympathetic nerve activity.^103^ Animal experiments have shown that Guasha has no effect on the morphology of local tissues and is safe and reliable.^104^ Spermatogenesis is regulated by the testicular microenvironment. Although there are no reports on scraping and spermatogenesis at present, this might be a direction for future research.

##### Cupping

The cupping was to use the tank to produce negative pressure, and then adsorb locally on the body surface where the pain and uncomfortable feeling happened, so as to treat the disease; its security was verified.^105^ In *TCM* theory, cupping stimulates the corresponding parts of the skin congestion and promotes the meridians or *Qi‐Blood* vigorous, promoting blood circulation and detumescence, relieving pain, dissipating wind and cold, or achieving other effects.^106^

Cupping has been reported to alter local immune function and activate neuroendocrine immune networks, enabling infertile women to conceive successfully.^107^ Cupping could improve perineal blood circulation and promote sperm production This filled us with anticipation and desires.

##### Tuina (therapeutic massage)

As an effective *TCM*, Tuina can achieve good results in the treatment of the nervous system,^108^ sleep,^109^ gastrointestinal diseases,^110^ dysmenorrhea,^111^ growth,^112^ cancer,^113^ and other diseases. The main mechanism is to promote local blood circulation through the use of point, dial, pressure, beat, roll, and other techniques by finger, palm, or elbow acupuncture on the meridians and acupoints, and to improve the overall function of the body.^99^, ^114^, ^115^

Similar to scraping and cupping, massage has rarely been reported in the treatment of male infertility. We hope that this will become a new direction for research.

##### Poultice

Poultice had become an effective way to treat skin diseases,^116^ injuries,^117^ and pain^118^ in many countries around the world.^119^, ^120^ It can act on the local drug more directly to solve the local problem.

Ancient Chinese doctors had learned to choose certain drugs, which combined with media (such as water, vinegar, wine, egg white and honey, vegetable oil, liquid medicine, or Vaseline ointment) to tune into a paste, pill, or cake and then stick on acupuncture points or the sore, to stimulate bullishness and regulate *Qi‐Blood* and *Yin‐Yang* to achieve health care and disease treatment, which was the mechanism of Poultice.^121^

There have been studies that treat late‐onset hypogonadism by converting testosterone into a poultice to obtain good results.^122^ This would be applicable for cryptorzoospermia, which would bring great benefits to patients.

##### Others

In addition to the above methods, *TCM* also includes T*ai‐Chi, Qi‐gong, yoga*, and other methods.^123^ These oriental medical strategies have unexpected effects on many diseases, such as cardiorespiratory fitness,^124^ rehabilitation after *COVID‐19*,^125^ cancer,^126^, ^127^ neurocognitive functions,^128^ Stroke^129^ and so on.

However, these traditional treatment methods have not been reported for cryptorzoospermia. With the development of modern science and its full combination with Western Medicine, these traditional methods will reflect their new vitality and become effective complementary treatments for male infertility in the future.

## 4 CONCLUSION

Although there was no universally recognized effective plan for the treatment of cryptorzoospermia, with the development of empirical treatments and clinical controlled trials, the effectiveness of cryptorzoospermia and *TCM* was gradually being verified by evidence‐based medicine. Especially the *TCM*, although, it had not been fully validated by evidence‐based medicine, but with the progress of combination with Western Medicine, its effectiveness and value were more and more accepted and affirmed in current days, which might be an effective supplement to Western Medicine, and some even had unexpected effects. In the anamnesis of the male patient, it is worth knowing if he takes TCM as some clinicians may forget to ask it, especially in Western countries.

### 4.1 Deficiencies and prospects

Due to the few clinical studies on the combination of Western Medicine and *TCM* were collected in this review, our views were still inadequate. More confirmatory studies and meta‐scores are needed to be confirm.

However, we are still full of hope and expectation for the future, because western medicine has focused on the development and application of artificial intelligence technology. With these means, TCM can also develop by leaps and bounds. The combination of western medicine and TCM will inevitably bring a more superior solution for cryptorzoospermia. It will surely become the nemesis of cryptorzoospermia and bring good news to infertile men worldwide.

## AUTHOR CONTRIBUTIONS

**Huang Liu**: Project design, literature analysis, and article writing. **Zefang Luo and Houbin Zheng**: Literature searching. **Jinghua Chen**: Illustration painting. **Qingqi Zeng**: Article guidance and revision.

## CONFLICT OF INTEREST

The authors declare no conflict of interest.

## ETHICS STATEMENT

Our study was approved by the Ethics Committee of Guangdong Reproductive Hospital (Approval Number: 2021[05]).

## TRANSPARENCY STATEMENT

The lead author Huang Liu, Qingqi Zeng affirms that this manuscript is an honest, accurate, and transparent account of the study being reported; that no important aspects of the study have been omitted; and that any discrepancies from the study as planned (and, if relevant, registered) have been explained.

## Data Availability

All the authors confirm that the data supporting the findings of this study are available within the article. Huang Liu had full access to all of the data in this study and takes complete responsibility for the integrity of the data and the accuracy of the data analysis.
